# XMPP for cloud computing in bioinformatics supporting discovery and invocation of asynchronous web services

**DOI:** 10.1186/1471-2105-10-279

**Published:** 2009-09-04

**Authors:** Johannes Wagener, Ola Spjuth, Egon L Willighagen, Jarl ES Wikberg

**Affiliations:** 1 Max von Pettenkofer-Institut, Ludwig-Maximilians-Universität, Munich, Germany; 2 Department of Pharmaceutical Biosciences, Uppsala University, Uppsala, Sweden

## Abstract

**Background:**

Life sciences make heavily use of the web for both data provision and analysis. However, the increasing amount of available data and the diversity of analysis tools call for machine accessible interfaces in order to be effective. HTTP-based Web service technologies, like the Simple Object Access Protocol (SOAP) and REpresentational State Transfer (REST) services, are today the most common technologies for this in bioinformatics. However, these methods have severe drawbacks, including lack of discoverability, and the inability for services to send status notifications. Several complementary workarounds have been proposed, but the results are ad-hoc solutions of varying quality that can be difficult to use.

**Results:**

We present a novel approach based on the open standard Extensible Messaging and Presence Protocol (XMPP), consisting of an extension (IO Data) to comprise discovery, asynchronous invocation, and definition of data types in the service. That XMPP cloud services are capable of asynchronous communication implies that clients do not have to poll repetitively for status, but the service sends the results back to the client upon completion. Implementations for Bioclipse and Taverna are presented, as are various XMPP cloud services in bio- and cheminformatics.

**Conclusion:**

XMPP with its extensions is a powerful protocol for cloud services that demonstrate several advantages over traditional HTTP-based Web services: 1) services are discoverable without the need of an external registry, 2) asynchronous invocation eliminates the need for ad-hoc solutions like polling, and 3) input and output types defined in the service allows for generation of clients on the fly without the need of an external semantics description. The many advantages over existing technologies make XMPP a highly interesting candidate for next generation online services in bioinformatics.

## Background

Life sciences make heavy use of the web as a medium for data access and computational analyses. Web services make such functionality available to machines while HTML-only web pages do not [1,2]. The ability to have two or more machines interact to solve bioinformatics problems opens up a wide area of computing, beyond the scope of manually visiting web pages. For example the members of the International Nucleotide Sequence Database Collaboration  and many other institutions offer public accessible and machine-readable services to retrieve, submit, or analyze bioinformatic data [3-6]. In recent years various tools have been introduced to aggregate services, among them workflow environments like Taverna [7] and Cyrille2 [8], and scripting environments like BioPipe [9]. This aggregation of services shows great potential, as exemplified in the successful experiment in [10] where microarray data, genomic sequence information, and pathway databases were integrated in a workflow to aid the search for candidate genes responsible for phenotypic variation.

The most common technologies underlying life science Web services are SOAP (Simple Object Access Protocol), BioMoby [11] and REST (Representational State Transfer). These technologies formalize how computers exchange messages, such as assignments, input data, computation results, and the output of database searches. SOAP and BioMoby are extensively used in life sciences [12,13], but the complexity and initial version incompatibilities of libraries that implement the SOAP specification have made REST an increasingly popular alternative [14]. While SOAP and BioMoby wrap the data in XML envelopes, REST can send around any data type. This makes REST less formal, easier to implement and use, but the lack of semantics also makes it difficult to write general clients for [1]. The Web Application Description Language (WADL) is intended to formalize REST services to overcome this issue [15] but is unfortunately not widely supported yet. In bioinformatics, BioMart [16] and DAS [17] are prominent examples of projects that utilizes the REST technology for data provision.

A problem in bioinformatics is that SOAP-based services, which are common in the field, suffer from various design flaws. Firstly, although the specification does not require this [18,19], SOAP services are typically using Hyper Text Transfer Protocol (HTTP) as communication channel. HTTP was originally designed to accommodate query and retrieval of web pages and is not aimed to more complex communication, something which is common in bioinformatics. The intrinsically synchronous HTTP protocol is unsuitable for time-consuming operations, like computationally demanding database lookups or calculations, and server timeouts and firewall issues are common obstacles. A very common workaround is to implement a ticketing mechanism in the service, where the client receives a ticket that can be used to repetitively poll for results. This is not only inefficient but also makes it impossible to create a general client for these services, as services implement the ticketing mechanism in a not standardized way. Secondly, SOAP services do not specify which data types are supported as input and output, which can be described with an XML Schema document. A separate specification, the Web Service Description Language (WSDL), similar to WADL, has been set up to address this, with the result that two technologies are required instead of only one. Thirdly and finally, the SOAP specification does not by itself provide means of service discovery, and another complementary technology, Universal Description Discovery and Integration (UDDI), has been developed for this purpose. To our knowledge UDDI is not used in bioinformatics.

BioMoby was more recently developed for life sciences as layer on top of SOAP to address its limitations, and allows for service annotation. It uses a data type ontology to describe which service supports which kind of life science data, taking the same role WSDL has, with users having the possibility to register new custom data types where necessary. BioMoby also has established a service registry, addressing the service discovery missing from SOAP itself. However, BioMoby still separates the services from their properties, and being restricted by the underlying HTTP protocol, it also does not support efficient asynchronous communication without repetitive polling. However BioMoby provides a standardized polling mechanism wich makes it currently better than plain SOAP-based services [20].

This paper introduces a method to overcome the limitations of currently available Web service technologies. It introduces a protocol (IO Data) that is based on top of the Extensible Messaging and Presence Protocol (XMPP) to overcome limitations of the HTTP transport protocol. The Web Services Glossary [21] defines "*[a] Web service is a software system designed to support interoperable machine-to-machine interaction over a network. It has an interface described in a machine-processable format (specifically WSDL). Other systems interact with the Web service in a manner prescribed by its description using SOAP-messages, typically conveyed using HTTP with an XML serialization in conjunction with other Web-related standards*". Even though they acknowledge that "*[t]here are many things that might be called 'Web services' in the world at large*", we will refer to the method proposed in this paper as *cloud services*, where the cloud is a synonym used for the cloud of resources available on the Internet [22].

**Figure 1:**
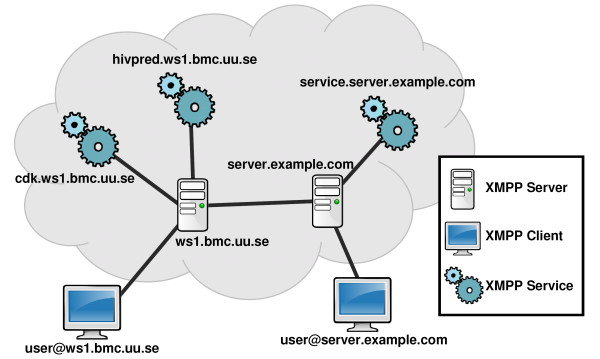
**XMPP cloud services connected to a federated network of XMPP servers**. The XMPP servers are taking care of routing XMPP stanzas from one XMPP entity to another inside the cloud. Each XMPP entity is identified by its Jabber ID (JID). XMPP services (e.g. hivpred.ws1.bmc.uu.se) are connected to an XMPP server (e.g. ws1.bmc.uu.se). XMPP clients (e.g. user@ws1.bmc.uu.se) connect to their XMPP server and can subsequently communicate with any XMPP entity connected to the federated network.

The Extensible Messaging and Presence Protocol (XMPP) is an open and decentralized XML routing technology that allows any entity to actively send XMPP messages to another entity [23]. The XMPP network consists of XMPP servers, clients, and services (see Figure 1). Each XMPP entity is identified by a unique identifier, the so called Jabber ID (JID). XMPP services are hosted by XMPP servers and offer remote functionality to other XMPP entities connected to the network, for example to XMPP clients. All traffic is routed through the XMPP servers. When required, e.g. users of different XMPP servers want to exchange messages, the involved XMPP servers initiate server-to-server connections, consequently forming a loose network. The XMPP server component that handles the server-to-server communication is the entry point to the public XMPP cloud. This entry point is configurable allowing the creation of private clouds. The conventional use of XMPP has been in instant messaging software like Jabber and Gmail chat, but a large collection of XMPP Extension Protocols (XEPs) extends the core specification, widening the scope of XMPP into various directions including remote computing. XMPP and HTTP are both used to transfer content from one computer to another. The main difference between the two protocols is how the messages are wrapped; whereas HTTP supports unstructured text, XMPP demands a pure XML environment, allowing seamless integration with the multitude of XML languages used in bio- and cheminformatics.

## Results

### XMPP Services

**Figure 2:**
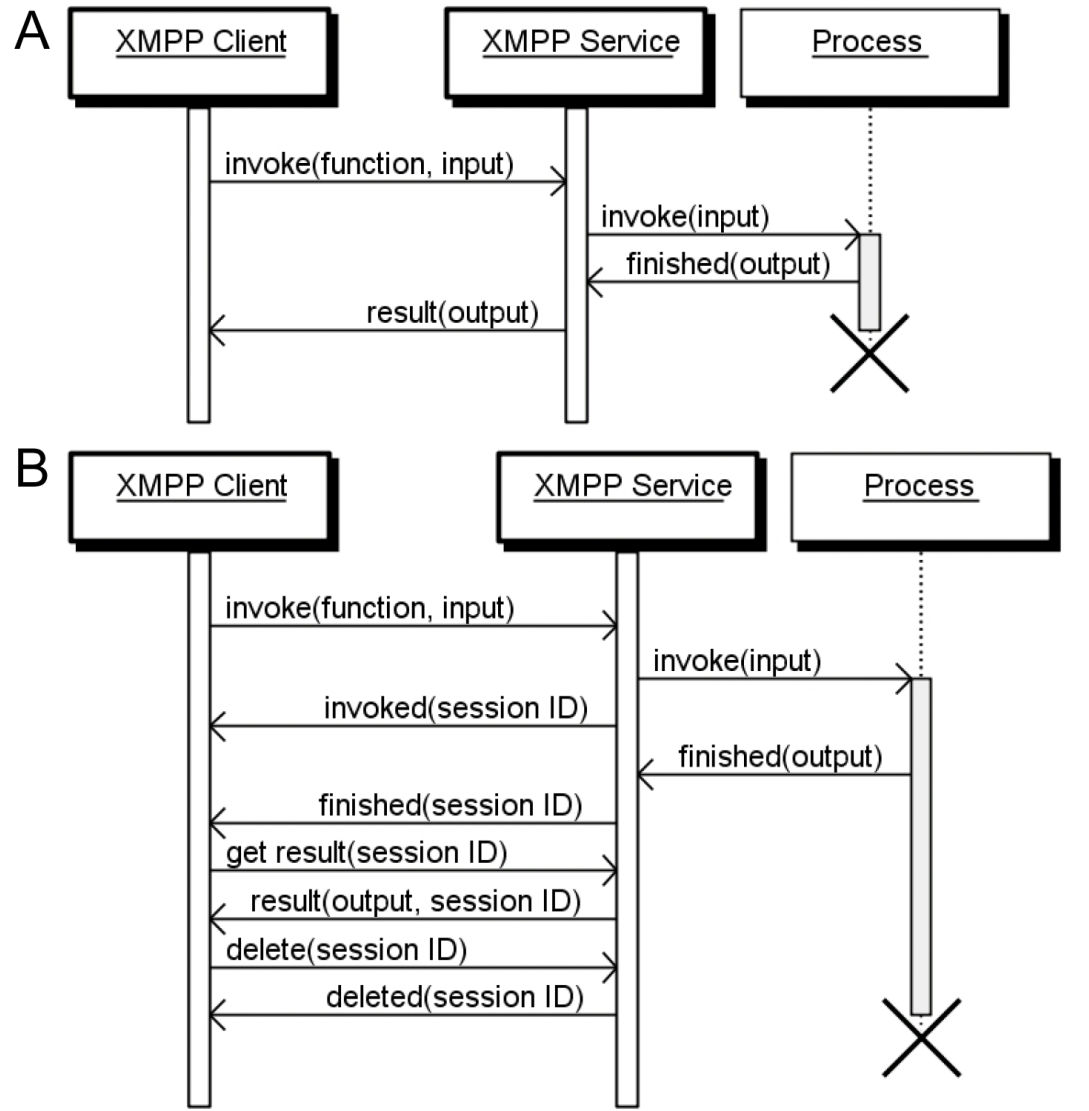
**Sequence diagrams for synchronous and asynchronous XMPP cloud services**. XMPP services may be implemented in the traditional synchronous way that directly returns the result when a method is invoked (A). Alternatively, services that perform time-consuming operations may be implemented in an asynchronous way where the client is notified after the initial job submission about the completion of the remote process, and the client subsequently requests the result from the service (B). The crosses indicate the end of a thread.

An XEP, named IO Data, was designed for sending messages from one computer to another, providing a transport for remote service invocation and attempting to overcome the problems with SOAP. The XEP was proposed as an experimental open standard to the XMPP Standards Foundation [24], and was later accepted as XEP-0244 [25]. This XEP solves two primary issues: 1) the unnecessary separation of the description (WSDL) and the actual SOAP service itself, and 2) asynchronous service invocation (see Figure 2). The XEP hence enables services to publish their own input and output data types, truly asynchronous invocation without any additional technology, and the advantage that existing XMPP infrastructure can be utilized to discover services [26].

### Reference Implementation

A reference implementation for the IO Data XEP, *XMPP Web Services for Java *(xws4j), was developed. The library is written in Java and provides a simple framework for developing and deploying XMPP cloud services and clients. Demonstration code to show how the library is used to generate and call services, as well as documentation, is provided together with the library's download packages and on the xws4j project web page [27].

To accommodate the large number of data types in life sciences, a library was developed (xws4j-binding) to create Java bindings and client stubs from the XML Schemata that can be obtained from XMPP cloud services. This way any XMPP service can be consumed and resulting data objects can directly be used in subsequent analysis, which is extremely powerful when pipelining services. The implementation is generic and works with any valid XML Schema. A specific XMPP cloud service, *XML Schemata Compatibility Service*, was set up to demonstrate binding generation for several XML Schemata in life sciences: the XML Schema definitions of EMBL Nucleotide Sequence Database XML [28], Functional Genomics Experiment [29], Genomic Contextual Data Markup Language [30], Molecular Interaction XML Format [31], Systems Biology Markup Language [32], and UniProt Knowledgebase [33].

### Cloud Services for Bio- and Cheminformatics

XMPP services were developed using xws4j for bioinformatics and cheminformatics, as shown in Table 1. A service, *HIVPred*, was set up that predicts the susceptibility of sequences for seven known HIV protease inhibitors using a proteochemometric model [34]. A Resource Description Framework service (*RDF Service*) has been added to show that RDF/XML integrated well, allowing integration in ongoing semantic web efforts in life sciences. Two XMPP services provide functionality to perform various cheminformatics algorithms (*CDK Service*), and to calculate various molecular descriptors (*Descriptor Service*) using the Chemistry Development Kit [35,36]. The *CDK Service *allows calculation of 2D and 3D coordinates and to derive various simple molecular properties for small molecules. Particularly the calculation of 3D coordinates can be time consuming for larger molecules and invoking those asynchronously is highly advised. The molecular descriptors returned by the *Descriptor Service *are numerical representations of the chemical structure which reflect some property of the structures; for example, it can describe the shape of the molecule, or the presence of certain functional groups, such as amine and acid groups. Calculating a single descriptor is typically fast, but calculating many descriptors for one molecule can be time consuming. The cheminformatics services apply the Chemical Markup Language (CML) [37] to define the services' semantics. CML is also used in various other applications and resources, and therefore provides a desirable interoperability with those resources. While CML is a fairly large XML Schema (137 elements, 217 attributes, 553 kB) [38-40], the xws4j library has no problem generating client side bindings on the fly.

**Table 1:** A list of XMPP services available from the XMPP server ws1.bmc.uu.se

**Name**	**Service JID**	**Description**
CDK Service	cdk.ws1.bmc.uu.se	The CDK Service was implemented using the Chemistry Development Kit (CDK) [35], which was previously done with SOAP [58] and local Taverna nodes [59].
Descriptor Service	descriptor.ws1.bmc.uu.se	The Descriptor Service was implemented using the QSAR module of the Chemistry Development Kit (CDK) [35]. The services provides four common descriptors TPSA, BCUT, Lipinski's Rule-of-Five, and the XLogP described in reference [36].
Blast Service	blast.ws1.bmc.uu.se	A proxy service for the SOAP-based blast service provided by the Center for Information Biology and DNA Data Bank of Japan
HIVPred	hivpred.ws1.bmc.uu.se	A service for predicting the susceptibility of a mutated HIV protease sequence for seven known HIV inhibitors based on a Proteochemometrical model [34] was implemented as an XWS service. The service takes a mutated HIV protease sequence of 99 amino acids as input and outputs numerical values and remarks on model validity, coverage, and applicability for the input.
XML Schemata Compatibility Service	bioschemata.ws1.bmc.uu.se	The XML Schemata Compatibility Service is a primitive demo XMPP service that delivers the following XML Schemata as function semantics: EMBL Nucleotide Sequence Database XML [28], Functional Genomics Experiment [29], Genomic Contextual Data Markup Language [30], Molecular Interaction XML Format [31], Systems Biology Markup Language [32], and UniProt Knowledgebase [33].
RDF Service	rdf.ws1.bmc.uu.se	The RDF Service shows how XMPP services can use RDF as IO format.

Complex calculations, e.g. which incorporate multiple database requests or simply are computationally intensive, will not finish within a few seconds after invocation. To demonstrate the suitability for time-consuming services the *Blast Service *was set up that supports asynchronous client notification. Although this is just a simulation - the *Blast Service *simply wraps an existing SOAP service provided by the Center for Information Biology and DNA Data Bank of Japan [6] - it shows the advantages of this approach: after the remote process is invoked the client waits for a notification message. After the notification message is received the client requests the result from the service. Several sample scripts were prepared that show how a result is retrieved after the calculation finished, and how the remote data is deleted after successful retrieval [see Additional files 1, 2, 3 and 4].

### Integration with Desktop Tools

**Figure 3:**
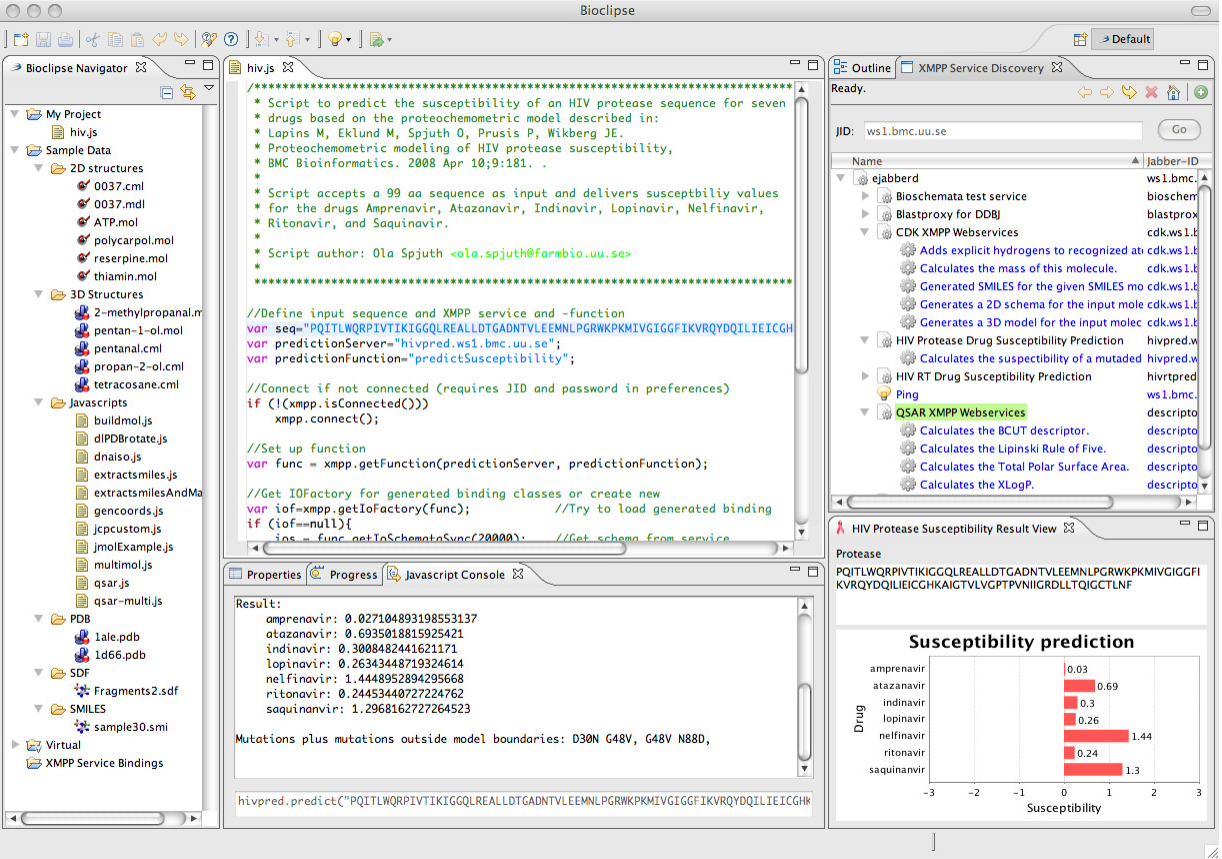
**XMPP services used from Bioclipse**. Screenshot from Bioclipse with the *HIVpred *plugin installed. The Bioclipse Console (bottom) was used to invoke the *HIVpred *service, and the results are displayed in this console and graphically in a chart (bottom right). In the top middle is shown a script making use of the *HIVpred *service for pipelined usage. The *Service Discovery View *displays the available services on the server ws1.bmc.uu.se (top right).

Bioclipse is a graphical workbench for life science with advanced integration and scripting capabilities [41]. Based on the xws4j library, two plugins were developed for Bioclipse. The first plugin provides the platform with generic XMPP service consumer functionality, facilitating discovery and invocation of XMPP services from the Graphical User Interface (GUI) as well as from the integrated scripting environment (see suppl. data for example scripts to call XMPP services). The *Service Discovery View *in Bioclipse is capable of listing XMPP services of any XMPP server, and services in turn are able to list their provided functions (see Figure 3). The client is able to discover changes in any XMPP entity, for example if a technical problem occurs and the entity is unavailable. Discovery of XMPP entities is not restricted to the local XMPP server the user is connected to but rather discovery of any XMPP entity is possible as long as the hosting XMPP server allows public access. As a consequence, an XMPP entity can also list XMPP entities that are not associated with the hosting server. The second Bioclipse plugin is a specific client for the HIVPred service that provides convenience methods as well as visualizes results in a bar diagram.

**Figure 4:**
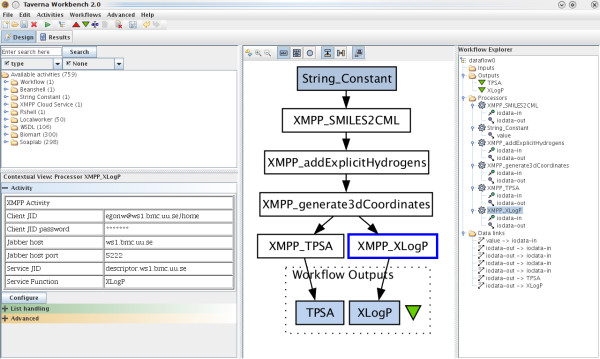
**XMPP plugin for Taverna**. Screenshot of Taverna running a cheminformatics workflow that calculates the XLogP and TPSA of a set of molecules represented by SMILES. Each *activity *is a call to a CDK-based XMPP service [57].

Taverna is a software tool for designing and executing workflows [7]. A Taverna plugin was written that allows calling XMPP services as part of workflows (see Figure 4). The plugin introduces an *activity *that can be configured with the XMPP account information used to connect to the XMPP network, and the details of the XMPP service being called. The *activity *passes its inputdirectly to the service, and the returned output is passed unchanged to the *activity*.

## Discussion

Today's bioinformatics with high throughput technologies and large and complex information sources requires not only interfaces to users but also programmatic interfaces to allow machines to access these services. XMPP and the developed extension IO Data improve greatly over existing remote service technologies in bioinformatics. Many services in bioinformatics take longer than just a few seconds to complete, for example querying over large or multiple databases, and computationally intensive calculations like protein structure prediction [42], identification of protein domains, families and functional sites [43], and sequence alignments [44]. These services would directly benefit from the XMPP, which makes all custom polling mechanisms, common with long running SOAP-based services, redundant. Such workarounds, called for example "ticketing" or "polling", are ad hoc solutions to a problem inherited from HTTP. XMPP services are intrinsically asynchronous, and completely eliminate this obstacle. This not only applies if the bioinformatics operation is long in itself, but could also be due to heavy load on the server and/or a large job queue.

It is important to make cloud services and their semantics publicly available so that users can discover and use them. While SOAP-based Web services require other methods to publish (e.g. UDDI), XMPP not only allows discovering of services out of the box but also supports determining their status and availability, which is shown with the *Service Discovery View *of Bioclipse (see Figure 3). XMPP services are connected to a single XMPP server, but since XMPP servers can communicate server-to-server it allows users to discover and consume services hosted on other servers. However, since new servers do not publish their availability to the XMPP network automatically, it is conceivable that a registry, similar to the recent initiatives like the Embrace Service Registry [45] and BioCatalogue [46], will be required to find new servers and their services. The already existing XMPP feature to run manually curated service directories makes this trivial [26]. However, we see the Embrace Service Registry and BioCatalogue as excellent complements and envision them to present XMPP services next to SOAP and REST services in the near future.

IO Data and SOAP both use XML Schema to describe a service's semantics, and in the last years several projects defined XML Schemata for data commonly used in life science, for example the Systems Biology Markup Language [32]. The *XML Schemata Compatibility Service *demonstrates that these XML Schemata for life science are compatible with XMPP. Reusing such generally accepted XML data types makes pipelining of services straightforward, as it is easy to attach a service with a certain output to another service that accepts this as input. This methodology of reusing certain defined data types has previously been shown successfully with BioMoby [11]. While BioMoby explicitly annotates a service with an entry from its data type ontology, XMPP services annotate themselves by publishing what XML Schema they support instead. This does not have the advantage that BioMoby puts on top of SOAP to have the data type independent from any schemata, however BioMoby's ontology does not allow a smooth integration of external formats, making reuse of emerging or existing standard formats like those used in the *XML Schemata Compatibility Service *difficult. In contrast to BioMoby where the common use of the centralized ontology guarantees the compatibility of different BioMoby services, the developer of an XMPP services must choose an adequate XML Schema himself, ideally using an emerging or existing standard. It is interesting to note that existing BioMoby services can be rewritten as XMPP services, requiring only the BioMoby data type to be expressed as XML Schemata.

However, while SOAP relies on a separate document (WSDL) to describe the service, IO Data makes this information available from the service itself. Removing an external file reduces the hassle to download a WSDL file from a web page. All the user needs to start is the JID of the XMPP server.

Service federation is a concept to address the need for trust agreements among decentralized security and policy domains, and is an important requirement for cloud service architectures [47]. Such scaffolds allow a commercial service provider to restrict access to registered customers, and non-public research groups to restrict access to collaborators. While HTTP-based Web services require the implementation of additional complex specification stacks, for example the WS-Security framework [48], the XMPP specifications describe authentication, security, and federation as core parts of the protocol [49,50], effectively causing all XMPP libraries to support such functionality by default: XMPP entities are primarily identified by their JIDs. As XMPP servers connect server-to-server on demand and form networks, with all traffic routed through these server-to-server connections, each XMPP client and service must authenticate with its server, and this authentication is easily controlled and/or extended by the server administrator. Additionally, XMPP servers support encrypted communication (SASL (Simple Authentication and Security Layer) and TLS (Transport Layer Security)) with the option to restrict XMPP servers to accept only encrypted client-to-server and server-to-server connections. All this implies that XMPP and IO Data are well suited for forming the basis of a federated service infrastructure.

Despite the many advantages of the XMPP cloud services there are some limitations when it comes to transfer of large data for two reasons. Firstly, though not restricted to XMPP, all data transmitted with XMPP must be wrapped in XML. This requires data conversions that might be less efficient when compared to a plain binary stream transmitted through a network connection. Secondly, each XMPP message must be submitted sequentially through the XMPP stream. In case a large message is transmitted the following messages are retained until the transmission of the large message completes. XMPP servers react on this issue by having a "maximum stanza size" that limits the accepted and processed message size. The default "maximum stanza size" differs from server software to server software and ranges from a few kilobytes to several megabytes. After adjusting the "maximum stanza size" in our test settings' server we experienced no problems transmitting messages of several megabytes of size. However this clearly shows that XMPP by itself is not the best choice for performing large file transfers. This limitation does not only concern cloud services but all other sorts of XMPP-based applications, too. For example instant messengers are often used to share files and to perform voice and video chat, both being bandwidth hungry. The XMPP community has solved this issue by defining extensions that are used to initiate out-of-band file transfers or voice over IP sessions. An XMPP service has the possibility to take advantage of such XMPP extensions, and "pass by reference" is currently explored by the authors as an extension to IO Data.

Encapsulating technical details is important to hide complexity from developers, while making it easier to write intuitive end user tools to take advantage of the possibilities of XMPP. This research has shown how XMPP services can be integrated using a Java library for XMPP into existing workflow and scripting frameworks like Taverna and Bioclipse. However, the Java library is only a reference implementation of the proposed protocols and it is our intention to integrate XMPP cloud services in other frameworks by delivering client and server libraries in other programming languages. There are a large number of open source XMPP libraries that can serve in this approach [51].

Finally, it is worth mentioning that reusing XMPP, which is a versatile transport protocol not primarily aimed at a single task, has many advantages. XMPP is already extended by a long list of other XEPs, defining other domains of application. Among these are extensions that bridge the XMPP network to other communication channels, such as the Short Message Service (SMS). This opens up a wide variety of new possibilities to easily integrate cloud services with human oriented communication. For example, we are exploring services to optionally send an SMS message when a time consuming calculation has finished.

## Conclusion

Web services are commonplace in bioinformatics now, but the currently used de facto standards HTTP, SOAP and WSDL make it a non-trivial task to set up, maintain, and publicly propagate a service. To overcome these issues, several solutions have been proposed. The EMBRACE consortium  has defined best practices for implementing services, and set up a website for service directory. The latter has also been addressed in the BioCatalogue and BioMoby registries. Additionally, BioMoby addressed the lack of strong data typing by defining an ontology of scientific data types, and annotation of services with these data types. The increasing use of REST services in bioinformatics also reflects the need for services that are easy to consume, but REST lacks the strong data typing needed in life sciences.

This paper introduces an alternative to SOAP-based Web services, providing intrinsic asynchronous capabilities, a tight integration with service discovery, and strong data typing by the service itself. The proposed cloud services use the versatile XMPP as the transport protocol, and the results have shown that the method can be used for a wide variety of bioinformatics tasks and how easily they can be integrated with existing scripting and workflow platforms for consuming remote services. Although development of HTTP started much earlier compared to XMPP (Jabber/XMPP was invented in 1998 by Jeremie Miller) we believe XMPP has reached a technical maturity and acceptance to be a robust and flexible basis for future cloud service technologies in bioinformatics.

## Methods

### XMPP

XMMP is the transport layer for the described method. The core protocols were formalized by the XMPP Working Group of the Internet Engineering Task Force [49,50]. XMPP Extension Protocols (XEPs) that formally define new functionality or features (where no adequate solution exists) are developed by the XMPP Standards Foundation (XSF) in an open standards process [24]. The IO Data extension (XEP-0244) is built on top of the Ad-Hoc Commands extension (XEP-0050) [25,52]. Service discovery is performed according to the specification of the Service Discovery extension (XEP-0030) [26].

### IO Data Implementation

The *XMPP Web Services for Java *(xws4j) library is written in Java and provides a Java framework for developing XMPP services and XMPP service clients [27]. The library depends on the JABBER Stream Objects (JSO-0.12.5) library [53] to make use of XMPP. The service provider is realized as a connecting XMPP server component that connects to an XMPP server via TCP/IP and is compatible with most current XMPP server implementations. The service consumer is realized as a standard XMPP client and requires a pre-existing XMPP account. The xws4j library optionally provides an additional tool to auto-generate a client stub for an XMPP service. This tool depends on the XMLBeans 2.4.0 library [54]. The xws4j library is compatible with Java 5.0 and higher.

### XMPP Server Implementation

The services presented here are running as components linking to an ejabberd 2.0 server [55] which is available under the domain and JID "ws1.bmc.uu.se". Settings were set to default except for the maximum stanza size for server-to-server connections and server-to-client connections, which were set to 32 and 16 MB respectively.

### The Chemistry Development Kit

The Chemistry Development Kit (CDK) is the open source library used for the cheminformatics functionality [35]. It provides algorithms for the calculation of 2D layouts as well as molecular descriptors for QSAR applications [36]. Services were implemented using CDK version 1.2.1. The Chemical Markup Language readers and writers are used for input and output [56].

### Bioclipse

Bioclipse is an open source, visual platform for chemo- and bioinformatics based on the Eclipse Rich Client Platform (RCP) [41]. The provided plugins were developed for Bioclipse 2.0. Recent developments in Bioclipse focused on extensive scripting support, to which plugins can contribute new commands.

### Taverna

Taverna is a free software tool for designing and executing workflows [7]. Taverna has plugin mechanism which allows third party developers to plug in additional functionality, such as *activity*. These *activities *are the technique by which the workflow provides input and retrieves output from the code for which this *activity *was written. The input and output ports of an *activity *are hard coded in the source code, and are linked to third party code to act on the input. After the computation has completed, the output is send to the output port and consumed by the rest of the workflow. Taverna 2.0 was used in this work.

## Availability and requirements

Project name: xws4j

Project home page: 

Operating system(s): platform independent 

Programming language: Java 

Other requirements: Java 1.5 or higher, JABBER Stream Objects (JSO-0.12.5),

XMLBeans 2.4.0

License: GNU Lesser General Public License 3

Any restrictions to use by non-academics: none

Project name: CDK-XWS services

Project home page: 

Operating system(s): platform independent

Programming language: Java

Other requirements: Java 1.5 or higher, JABBER Stream Objects (JSO-0.12.5), xws4j,

The Chemistry Development Kit 1.2.1 

License: GNU Lesser General Public License 2.1

Any restrictions to use by non-academics: none

Project name: XMPP-Taverna Plugin 

Project home page: 

Operating system(s): platform independent

Programming language: Java

Other requirements: Java 1.5 or higher, Taverna 2.0

License: GNU Lesser General Public License 3

Any restrictions to use by non-academics: none

Project name: Bioclipse XMPP Plugins

Project home page: 

Operating system(s): platform independent

Programming language: Java

Other requirements: Java 1.5, Bioclipse 2.0

License: Eclipse Public License

Any restrictions to use by non-academics: none

There is a wiki with articles, tutorials, services, sample scripts, and more information about XMPP Services available at .

## Authors' contributions

JW designed the XEP, implemented the xws4j and xws4j-binding libraries, and constructed the XMPP plugin for Bioclipse. OS implemented the *HIVPred *service, the *HIVPred *plugin for Bioclipse, and tested all implementations. ELW implemented the *CDK*, *QSAR *and *RDF Services*, as well as the Taverna 2 plugin. All authors were involved in manuscript preparation. All authors read and approved the final manuscript.

## Supplementary Material

Additional file 1 **Sample script demonstrating the invocation of an XMPP service**. This script demonstrates the synchronous (blocking) API of xws4j used from the Bioclipse javascript environment. The invoked function (calculateMass) finishes within seconds. Click here for file

Additional file 2 **Sample script demonstrating the simple invocation of a time-consuming XMPP service**. This script demonstrates the listener-based asynchronous API of xws4j used from the Bioclipse javascript environment. It may take several minutes to complete. Click here for file

Additional file 3 **Sample script demonstrating the invocation of a time-consuming XMPP service**. This script demonstrates the listener-based asynchronous API of xws4j used from the Bioclipse javascript environment. It may take several minutes to complete. With the session ID returned from the service (see onExecuting() in the listener) one may check the status or terminate the remote process. Click here for file

Additional file 4 **Sample script demonstrating the abortion of a time-consuming XMPP service**. This script demonstrates how to continue/pick and terminate the blast that was initially invoked with the script 'invokeblast.js' (see Additional file 3). To pick an existing remote process its session ID must be specified. Click here for file

## References

[B1] Stockinger H, Attwood T, Chohan SN, Côté R, Cudré-Mauroux P, Falquet L, Fernandes P, Finn RD, Hupponen T, Korpelainen E, Labarga A, Laugraud A, Lima T, Pafilis E, Pagni M, Pettifer S, Phan I, Rahman N (2008). Experience using web services for biological sequence analysis. Brief Bioinform.

[B2] Stein L (2002). Creating a bioinformatics nation. Nature.

[B3] Sayers EW, Barrett T, Benson DA, Bryant SH, Canese K, Chetvernin V, Church DM, DiCuccio M, Edgar R, Federhen S, Feolo M, Geer LY, Helmberg W, Kapustin Y, Landsman D, Lipman DJ, Madden TL, Maglott DR, Miller V, Mizrachi I, Ostell J, Pruitt KD, Schuler GD, Sequeira E, Sherry ST, Shumway M, Sirotkin K, Souvorov A, Starchenko G, Tatusova TA, Wagner L, Yaschenko E, Ye J (2009). Database resources of the National Center for Biotechnology Information. Nucleic Acids Res.

[B4] Miyazaki S, Sugawara H, Ikeo K, Gojobori T, Tateno Y (2004). DDBJ in the stream of various biological data. Nucleic Acids Res.

[B5] Labarga A, Valentin F, Anderson M, Lopez R (2007). Web services at the European bioinformatics institute. Nucleic Acids Res.

[B6] Sugawara H, Miyazaki S (2003). Biological SOAP servers and web services provided by the public sequence data bank. Nucleic Acids Res.

[B7] Oinn T, Addis M, Ferris J, Marvin D, Senger M, Greenwood M, Carver T, Glover K, Pocock MR, Wipat A, Li P (2004). Taverna: a tool for the composition and enactment of bioinformatics workflows. Bioinformatics.

[B8] Fiers MWEJ, Burgt A van der, Datema E, de Groot JCW, van Ham RCHJ (2008). High-throughput bioinformatics with the Cyrille2 pipeline system. BMC Bioinformatics.

[B9] Hoon S, Ratnapu KK, Chia J, Kumarasamy B, Juguang X, Clamp M, Stabenau A, Potter S, Clarke L, Stupka E (2003). Biopipe: a flexible framework for protocol-based bioinformatics analysis. Genome Res.

[B10] Fisher P, Hedeler C, Wolstencroft K, Hulme H, Noyes H, Kemp S, Stevens R, Brass A (2007). A systematic strategy for large-scale analysis of genotype phenotype correlations: identification of candidate genes involved in African trypanosomiasis. Nucleic Acids Res.

[B11] Wilkinson MD, Links M (2002). BioMOBY: an open source biological web services proposal. Brief Bioinform.

[B12] Neerincx PBT, Leunissen JAM (2005). Evolution of web services in bioinformatics. Brief Bioinform.

[B13] BioMoby Consortium (2008). Interoperability with Moby 1.0--it's better than sharing your toothbrush!. Brief Bioinform.

[B14] Jain E, Phan I, Duvaud S, Gasteiger E, Redaschi N, Martin MJ, McGarvey P, Bairoch A (2008). Design and Implementation of the UniProt Website. Nature Precedings.

[B15] Web Application Description Language. https://wadl.dev.java.net/.

[B16] Smedley D, Haider S, Ballester B, Holland R, London D, Thorisson G, Kasprzyk A (2009). BioMart--biological queries made easy. BMC Genomics.

[B17] Dowell RD, Jokerst RM, Day A, Eddy SR, Stein L (2001). The distributed annotation system. BMC Bioinformatics.

[B18] SOAP Over Java Message Service 1.0. http://www.w3.org/TR/2008/WD-soapjms-20081121.

[B19] SOAP Version 1.2 Email Binding. http://www.w3.org/TR/2002/NOTE-soap12-email-20020703.

[B20] Web Services Resource 1.2 (WS-Resource). http://docs.oasis-open.org/wsrf/wsrf-ws_resource-1.2-spec-os.pdf.

[B21] Web Services Glossary. http://www.w3.org/TR/2004/NOTE-ws-gloss-20040211/.

[B22] Vaquero LM, Rodero-Merino L, Caceres J, Lindner M (2009). A break in the clouds: towards a cloud definition. SIGCOMM Comput Commun Rev.

[B23] About XMPP. http://xmpp.org/about/.

[B24] XMPP Standards Foundation. http://xmpp.org/xsf/.

[B25] XEP-0244: IO Data. http://xmpp.org/extensions/xep-0244.html.

[B26] XEP-0030: Service Discovery. http://xmpp.org/extensions/xep-0030.html.

[B27] XMPP Web Services for Java. http://xws4j.sourceforge.net/.

[B28] The Second Stable Version of the EMBL Nucleotide Sequence Database XML Schema. http://www.ebi.ac.uk/embl/schema/EMBL_Services_V1.1.xsd.

[B29] Jones AR, Miller M, Aebersold R, Apweiler R, Ball CA, Brazma A, Degreef J, Hardy N, Hermjakob H, Hubbard SJ, Hussey P, Igra M, Jenkins H, Julian RKJ, Laursen K, Oliver SG, Paton NW, Sansone S, Sarkans U, Stoeckert CJJ, Taylor CF, Whetzel PL, White JA, Spellman P, Pizarro A (2007). The Functional Genomics Experiment model (FuGE): an extensible framework for standards in functional genomics. Nat Biotechnol.

[B30] Kottmann R, Gray T, Murphy S, Kagan L, Kravitz S, Lombardot T, Field D, Glöckner FO (2008). A standard MIGS/MIMS compliant XML Schema: toward the development of the Genomic Contextual Data Markup Language (GCDML). OMICS.

[B31] Kerrien S, Orchard S, Montecchi-Palazzi L, Aranda B, Quinn AF, Vinod N, Bader GD, Xenarios I, Wojcik J, Sherman D, Tyers M, Salama JJ, Moore S, Ceol A, Chatr-Aryamontri A, Oesterheld M, Stümpflen V, Salwinski L, Nerothin J, Cerami E, Cusick ME, Vidal M, Gilson M, Armstrong J, Woollard P, Hogue C, Eisenberg D, Cesareni G, Apweiler R, Hermjakob H (2007). Broadening the horizon--level 2.5 of the HUPO-PSI format for molecular interactions. BMC Biol.

[B32] Hucka M, Finney A, Sauro HM, Bolouri H, Doyle JC, Kitano H, Arkin AP, Bornstein BJ, Bray D, Cornish-Bowden A, Cuellar AA, Dronov S, Gilles ED, Ginkel M, Gor V, Goryanin II, Hedley WJ, Hodgman TC, Hofmeyr J, Hunter PJ, Juty NS, Kasberger JL, Kremling A, Kummer U, Le Novère N, Loew LM, Lucio D, Mendes P, Minch E, Mjolsness ED, Nakayama Y, Nelson MR, Nielsen PF, Sakurada T, Schaff JC, Shapiro BE, Shimizu TS, Spence HD, Stelling J, Takahashi K, Tomita M, Wagner J, Wang J (2003). The systems biology markup language (SBML): a medium for representation and exchange of biochemical network models. Bioinformatics.

[B33] UniProt Consortium (2009). The Universal Protein Resource (UniProt) 2009. Nucleic Acids Res.

[B34] Lapins M, Eklund M, Spjuth O, Prusis P, Wikberg JES (2008). Proteochemometric modeling of HIV protease susceptibility. BMC Bioinformatics.

[B35] Steinbeck C, Han Y, Kuhn S, Horlacher O, Luttmann E, Willighagen E (2003). The Chemistry Development Kit (CDK): an open-source Java library for Chemo- and Bioinformatics. J Chem Inf Comput Sci.

[B36] Steinbeck C, Hoppe C, Kuhn S, Floris M, Guha R, Willighagen EL (2006). Recent developments of the chemistry development kit (CDK) - an open-source java library for chemo- and bioinformatics. Curr Pharm Des.

[B37] Murray-Rust P, Rzepa HS (1999). Chemical Markup, XML, and the Worldwide Web. 1. Basic principles. J Chem Inf Comput Sci.

[B38] Holliday GL, Murray-Rust P, Rzepa HS (2006). Chemical markup, XML, and the world wide web. 6. CMLReact, an XML vocabulary for chemical reactions. J Chem Inf Model.

[B39] Kuhn S, Helmus T, Lancashire RJ, Murray-Rust P, Rzepa HS, Steinbeck C, Willighagen EL (2007). Chemical Markup, XML, and the World Wide Web. 7. CMLSpect, an XML vocabulary for spectral data. J Chem Inf Model.

[B40] Adams N, Winter J, Murray-Rust P, Rzepa HS (2008). Chemical Markup, XML and the World-Wide Web. 8. Polymer Markup Language. J Chem Inf Model.

[B41] Spjuth O, Helmus T, Willighagen EL, Kuhn S, Eklund M, Wagener J, Murray-Rust P, Steinbeck C, Wikberg JES (2007). Bioclipse: an open source workbench for chemo- and bioinformatics. BMC Bioinformatics.

[B42] Wallner B, Larsson P, Elofsson A (2007). Pcons.net: protein structure prediction meta server. Nucleic Acids Res.

[B43] Hunter S, Apweiler R, Attwood TK, Bairoch A, Bateman A, Binns D, Bork P, Das U, Daugherty L, Duquenne L, Finn RD, Gough J, Haft D, Hulo N, Kahn D, Kelly E, Laugraud A, Letunic I, Lonsdale D, Lopez R, Madera M, Maslen J, McAnulla C, McDowall J, Mistry J, Mitchell A, Mulder N, Natale D, Orengo C, Quinn AF, Selengut JD, Sigrist CJA, Thimma M, Thomas PD, Valentin F, Wilson D, Wu CH, Yeats C (2009). InterPro: the integrative protein signature database. Nucleic Acids Res.

[B44] Larkin MA, Blackshields G, Brown NP, Chenna R, McGettigan PA, McWilliam H, Valentin F, Wallace IM, Wilm A, Lopez R, Thompson JD, Gibson TJ, Higgins DG (2007). Clustal W and Clustal X version 2.0. Bioinformatics.

[B45] The EMBRACE Service Registry. http://www.embraceregistry.net/.

[B46] BioCatalogue: Providing a Curated Catalogue of Life Science Web Services. http://biocatalogue.org/.

[B47] Kaarthik S, Kunal V, Amit S (2004). Discovery of Web Services in a Federated Registry Environment. Proceedings of the IEEE International Conference on Web Services.

[B48] OASIS Web Service Security (WSS) TC. http://www.oasis-open.org/committees/wss.

[B49] Extensible Messaging and Presence Protocol (XMPP): Core. http://www.ietf.org/rfc/rfc3920.txt.

[B50] Extensible Messaging and Presence Protocol (XMPP): Instant Messaging and Presence. http://www.ietf.org/rfc/rfc3921.txt.

[B51] XMPP Software: Libraries. http://xmpp.org/software/libraries.shtml.

[B52] XEP-0050: Ad-Hoc Commands. http://xmpp.org/extensions/xep-0050.html.

[B53] JABBER Stream Objects. https://jso.dev.java.net/.

[B54] XMLBeans. http://xmlbeans.apache.org/.

[B55] Ejabberd - the Erlang Jabber/XMPP Daemon. http://www.ejabberd.im/.

[B56] Willighagen E (2001). Processing CML conventions in Java. Internet J Chem.

[B57] Workflow Entry: Run an XMPP Cloud Service. http://www.myexperiment.org/workflows/631?version=2.

[B58] Dong X, Gilbert KE, Guha R, Heiland R, Kim J, Pierce ME, Fox GC, Wild DJ (2007). Web service infrastructure for chemoinformatics. J Chem Inf Model.

[B59] CDK-Taverna. http://www.cdk-taverna.de/.

